# Characteristics of population structure, antimicrobial resistance, virulence factors, and morphology of methicillin-resistant *Macrococcus caseolyticus* in global clades

**DOI:** 10.1186/s12866-022-02679-8

**Published:** 2022-11-05

**Authors:** Yu Zhang, Shengyi Min, Yuxuan Sun, Jiaquan Ye, Zhemin Zhou, Heng Li

**Affiliations:** Pasteurien College, Suzhou Medical College, Soochow University, Suzhou, Jiangsu China

**Keywords:** *Macrococcus caseolyticus*, Retail meat, MLST type, Antibiotic resistance, Virulence genes

## Abstract

*Macrococcus caseolyticus* is an opportunistic pathogen that is frequently isolated from dairy products and veterinary infections. Recent studies have reported the possibility of methicillin resistance that be transferred among staphylococcal species in foods. The present study examined the population structure, antimicrobial resistance, virulence factors, and morphology of methicillin-resistant *M. caseolyticus* by investigation of 94 genomes derived from both isolates in beef (*n* = 7) and pork (*n* = 2) at Shanghai and those deposited in public domain (*n* = 85). Phylogenetically, *M. caseolyticus* were divided into four clades, which each consisted of genomes isolated from continent of European countries (82.4%, *n* = 78), Asian countries (11.3%, *n* = 10), United States (4.1%, *n* = 4), Australia (1%, *n* = 1), and Sudan (1%, *n* = 1). The *M. caseolyticus* isolated from present study formed a genetically distinguished clade, which was characterized by novel alleles in the traditional 7-gene MLST scheme. Furthermore, we identified 24 AMR genes that were associated with 10 classes of antimicrobial agents in *M. caseolyticus*. Most AMR genes were carried by dominant plasmids such as rep7a, rep22 and repUS56. The genomes in the global clades carried significantly less AMR genes (*p* < 0.05) and more virulence factors (*p* < 0.001) than present clade. Virulence factors were detected in methicillin resistant *M. caseolyticus* including genes coding hemolysin, adherence, biofilm formation, exotoxin, and capsule that associated to human health and infection. Finally, as the close relative of the genus *Staphylococcus*, scanning electron microscopy (SEM) and transmission electron microscopy (TEM) were performed for morphological comparison that *M. caseolyticus* has a larger diameter and thicker cell wall compared with *S. aureus* ATCC 25,923. Taken together, our study suggested that *M. caseolyticus* mediating divergent antimicrobial resistance and virulence factors could serve as the vector for methicillin resistance habitats in foodborne microorganisms.

**Supplementary Information:**

The online version contains supplementary material available at 10.1186/s12866-022-02679-8.

## Highlights

• The global lineage of *M. caseolyticus* strains were divided into four clades from A to D.

• MLST typing revealed novel alleles in *M. caseolyticus* strains isolated in China.

• Global clades carried significantly less AMR genes and more virulence factors than present isolates.

• As the close relative of the genus *Staphylococcus, Macrococcus caseolyticus *has a larger diameter and thicker cell wall compared with *S.aureus* ATCC 25923.

• *Macrococcus caseolyticus* may serve as a vector for methicillin resistance habitats in foodborne microorganisms.

**Supplementary Information:**

The online version contains supplementary material available at 10.1186/s12866-022-02679-8.

## Introduction

Initially described in 1916, *Macrococcus* are catalase-positive cocci that currently consist of 12 species, including *Macrococcus caseolyticus*, *Macrococcus bovicus*, *Macrococcus canis,* and many more [9, 25]. As the close relative of the genus *Staphylococcus, M. caseolyticus* exhibits a high homology in phenotypical and biological characteristics, and shares similarities with these oxidase-positive and novobiocin-resistant staphylococci [25]. Previously, *M. caseolyticus* was classified as *Staphylococcus caseolyticus* due to the high similarities [5, 7, 29], indicating the necessity to distinguish these two bacteria from a perspective of morphological, phenotypic, and genotypic approaches.

*Macrococcus caseolyticus* is an exotic bacterium that is frequently found in fermented cheeses, sausages, pigs, calves, pork, and beef meat in Europe [17]. It can enhance the flavor of dairy products by producing amino acid and lipid-derived flavor compounds [10, 15, 26]. However, the adaptive acquisition of methicillin resistance genes in *M. caseolyticus* genomes, such as *mecABCD* has been observed over the past decades [11, 28–30]. Comparing to classical *mecA* and *mecC* that are mostly carried by *Staphylococcus aureus,* the methicillin-resistant genes in *M. caseolyticus* are *mecB* and *mecD* respectively. Similar to the mechanism in methicillin-resistant *S. aureus* (MRSA), the *mec* complex in *M. caseolyticus* JCSC7096 is also associated with a transposon Tn6045, which can be horizontally transferred into other species in the *Macrococcus* genus [12].

Recent studies have reported high mortality rates in animals infected with Macrococci [4, 5, 20]. For instance, the *M. caseolyticus* SDLY strain isolated from commercial chickens exhibited severe pathogenicity, including hemorrhages and multifocal necrosis, which might be associated with the insertion of capsular polysaccharide synthesis genes in a virulence background [20].

To further characterize antimicrobial resistance, virulence factors, and morphology in global lineage of methicillin-resistant *M. caseolyticus*, a combined analysis of phenotypic, genomic, and morphological approaches was administrated on a set of 94 M*. caseolyticus* isolates. We examined the antimicrobial resistance and virulence factors in a global scale and described morphological comparison between *M. caseolyticus* and its close relative, *Staphylococcus aureus*, which allowed a better understanding of these two bacteria species.

## Method

### Sampling and collection

Samples from each of 24 outlets, including 11 in wet markets and 13 in the supermarkets, were collected between August and October of 2021 in Shanghai (Supply Table 1). The sampled outlets were distributed across 11 districts from Shanghai city, including Pudong (*n* = 6), Huangpu (*n* = 3), Xuhui (*n* = 2), Jing’an (*n* = 2), Minhang (*n* = 2), Yangpu (*n* = 2), Changning (*n* = 2), Putuo (*n* = 2), Songjiang (*n* = 1), Fengxian (*n* = 1), and Baoshan (*n* = 1). Packages of frozen chicken, beef, and pork were purchased from the outlets and transported to the laboratory on ice containers within 4 h of collection. Retail meat products were prepared for analysis as previously described [21]. Briefly, 10 g of samples were homogenized in 0.1% peptone saline in a filter bag (Bkmam, Changde, China) and 100 ul were cultured onto CHROMagar™ MRSA agar (Becton Dickinson, Franklin Lakes, NJ) for selection overnight at 37 °C [17]. The obtained *M. caseolyticus* strains were confirmed by 16 s rRNA sequencing and MALDI-TOF MS (Bio-M ´erieux, Craponne, France). Detail isolation and species confirmation were described in Supply Table 1.

### Phenotypic antimicrobial susceptibility test

Confirmed *M. caseolyticus* strains were enrolled for antimicrobial susceptibility test by disk diffusion (Oxoid) according to the guideline of Clinical and Laboratory Standards Institute (CLSI 2017). The antimicrobial compounds included ampicillin (10 μg), amikacin (30 μg), cefazolin (30 μg), cefuroxime (30 μg), ceftriaxone (30 μg), ceftazidime (30 μg), cefoperazone (75 μg), doxycycline (30 μg), erythromycin (15 μg), gentamicin (10 μg), kanamycin (30 μg), lincomycin (2 μg), minocycline (30 μg), penicillin (10 μg), piperacillin (100 μg), streptomycin (10 μg), tetracycline (30 μg), and with cefoxitin as methicillin-resistant drug control. *Escherichia coli* ATCC 25,922 and *S. aureus* ATCC 25,923 were used as the quality control.

### Whole genome sequencing

Nine *M. caseolyticus* strains isolated from this study were sent for DNA purification using HiPure Bacterial DNA Kit (D3146, Meiji Biotechnology Co., Ltd, Guangzhou, China) and sequenced via Illumina’s NextSeq 500 (Illumina, San Diego, CA, United States) platform of Honsunbio company (Shanghai, China).

### Public data retrieval and analysis

A total of 87 SRA genomes of *M. caseolyticus* were evaluated from GenBank (accessed in December 2021). After checking the duplicate samples, 51 SRA genomes were excluded, and addition two strains were removed due to the low identity by species identification. Finally, a total of 85 global strains were downloaded from GenBank including 34 sets of filtered SRA genomes and 51 already assemblies (Supply Table 2). All the raw reads were trimmed and assembled by EToKi, and quality was evaluated with QUAST v2.3 [13, 31]. This resulted to a global collection of genomes of 94 M*. caseolyticus* strains including present isolates. The raw reads from this study are submitted to the China National GenBank under project accession ID CNP0002826.

### Genomic analysis and SNP tree construction

All 94 M*. caseolyticus* assemblies were submitted for MLST typing (https://pubmlst.org) and screened for antimicrobial-resistant genes and virulence factors using online tools of ResFinder, MobileElementFinder, and VFDB [3, 14, 22]. Alignments with a minimum of ≥ 60% nucleotide identity was kept in all three programs, respectively. The single nucleotide polymorphism (SNP) tree was constructed using CSI Phylogeny v1.4 (http://genomicepidemiology.org/) by aligning other genomes onto a reference genomic sequence from *Macrococcus* sp. (ASM2009413).

### Phylogenetic tree with temporal and geographical datasets

EToKi was used to evaluate the 94 M*. caseolyticus* genomes with ASM211982 selected as the reference genome due to the highest N50 value [31]. Then EToKi align was applied to generate a matrix file to further build the phylogenetic tree containing the datasets of geography and years [31]. Afterwards, RecHMM and RecFilter were enrolled to filter the reorganization of the phylogenetic tree [31]. Finally, the dated tree was generated by using BactDating v1.1, and was then used to infer geographic distributions using treetime v1.0 [8, 27].

### Data visualization and statistical analysis

The genotypic data were visualized in Grapetree and iTOL v4.0 [19, 32]. The violin graph was drawn in GraphPad Prism 7 and statistical significance was calculated using One-way ANOVA with * *p* < 0.05, ** *p* < 0.01, **** *p* < 0.001. Description of antimicrobial-resistant genes and MLST were list in Supply Table 3 and Supply Table 4.

### Comparative morphological observation

To better understand the morphological differences between *M. caseolyticus* and its closest relative of *S. aureus*, the HB024567 strain from present study and *S. aureus* ATCC 25,923 were employed for SEM and TEM analysis. Prior to scanning and transmission electron microscope (SEM/TEM), the *M. caseolyticus* HB024567 and *S. aureus* ATCC 25,923 cultures were grown in 50 ml of LB at 37 °C with a starting OD600 of 0.02. Cells were harvested at OD600 ~ 0.5 and suspended in fixation solution and incubated overnight at 4 °C. After the treatments, cell pellets were washed twice with cacodylate buffer (0.05 M, pH 7.4) and post-fixed with 2% osmium tetroxide, followed by 0.25% uranyl acetate for contrast enhancement. The pellets were dehydrated with ethanol (30, 50, 70, 80, 90, and 100%), embedded in Epoxy resin, and cut into ultrathin sections for lead citrate staining. The final sections were examined by Philips CM100 BioTWIN transmission electron microscope. For the SEM, the suspended cells after post-fixation and dehydration were placed on stubs and coated with gold–palladium for 2 min. Then samples were observed by JSM-7500F scanning electron microscope (JEOL Ltd., Tokyo, Japan).

## Results

### Geographic distribution, population structure, and years of isolation

**Fig. 1:**
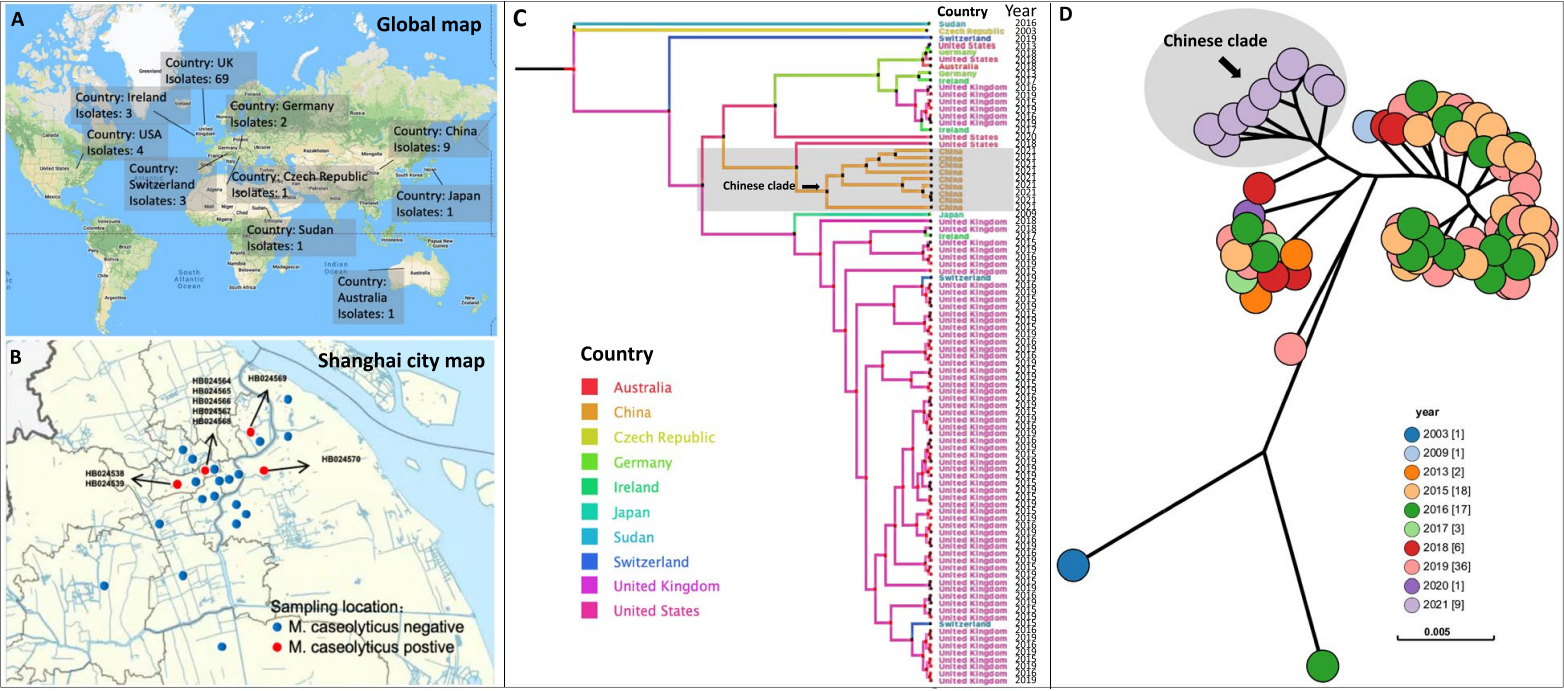
Characteristics of geographic distribution, phylogenetic analysis, and years of isolation for the *M. caseolyticus* strains. **A** Global distribution of 94 M*. caseolyticus* isolates from various countries. **B** Geographical distribution of nine *M. caseolyticus* isolated from beef and pork meat in Shanghai, China. **C** Phylogenetic analysis and spread prediction of 94 global *M. caseolyticus* isolates based on genome and country information. Grey box indicated preset isolates in this study. **D** Information on the isolation year of 94 global *M. caseolyticus* isolates. Grey oval indicated nine Chinese strains isolated in Shanghai, 2021

**Fig. 2:**
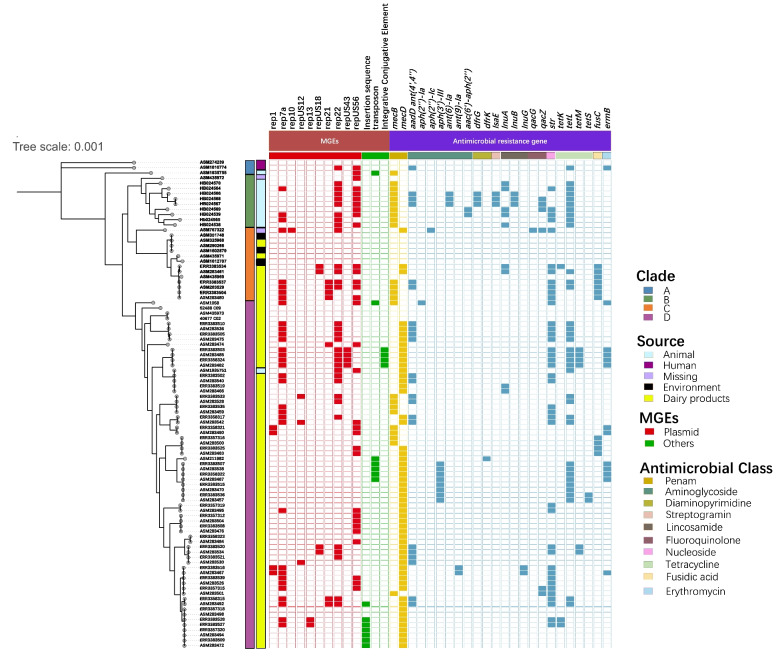
Distributions of MGEs and AMR genes in global *M. caseolyticus* clades. All *M. caseolyticus* strains were divided into four clades including A, B, C, and D based on phylogenetic tree. The squares colored by trait category represented the presence of MGE or AMR genes

A total of 94 M*. caseolyticus* strains were selected for geographic analysis including nine strains isolated from beef (*n* = 7) and pork (*n* = 2) in Shanghai and additional 85 isolates from GenBank (Fig. 1A/B). All strains were distributed in five continents including European countries (82.4%, *n* = 78), Asian countries (11.3%, *n* = 10), United States (4.1%, *n* = 4), Australia (1%, *n* = 1), and Sudan (1%, *n* = 1) (Fig. 1A/B). The phylogenetic tree showed that global *M. caseolyticus* strains were divided into four clades from A to D. Clade A formed two independent clusters that were separated from the rest strains and this clade was associated with human infection, while clade B from this study was mainly detected in retail meat (beef, *n* = 7; pork, *n* = 2) as a local Chinese cluster. Clade C and D had the dominant proportion isolated from bulk milk (Fig. 1C & 2). MLST typing of clade B showed the presence of novel alleles among the present *M. caseolyticus* strains, *e.g., cpn60, fdh, pta, purA* in HB024539 and *cpn60* and *pta* in HB024569 (Table 1 & Supp. Table 4). Afterwards, the years of isolation were assessed for all global *M. caseolyticus* strains. The present strains formed as a separated clade that isolated in 2021 (Gray oval, Fig. 1D), whiles the remaining strains showed diverse years of collection between 2003 and 2020 (Fig. 1D).

**Table 1:** Multi-locus sequence typing (MLST) of nine *M. caseolyticus* strains isolated from beef and pork meat in Shanghai, China

Strains	MLST^a^	ack	cpn60	fdh	pta	purA	sar	tuf
HB024538	18/53	6	2	9	5^b^	3	7	4
HB024539	18/53	6	13^b^	9^b^	10^b^	3^b^	7	5
HB024564	18/53	6	13^b^	9^b^	5	20^b^	7	5
HB024565	62	6	2	9^b^	10^b^	20^b^	17^b^	5
HB024566	18/53/62	6	2^b^/12^b^	9^b^	5	20^b^	1	5
HB024567	18/53/62	6	2^b^/12^b^	9^b^	5	20^b^	1^b^	5
HB024568	18/53	6	2^b^	9^b^	5	20^b^	2^b^	5
HB024569	3/31/32/33/62	6	3^b^	3	1^b^	3	1	4
HB024570	18/53	6	3^b^	9^b^	5	2	1	5

^a^ MLST was presented with the closest types

^b^ Novel alleles were indicated with the closest alleles numbers

### Identification of AMR genes and mobile elements

**Fig. 3:**
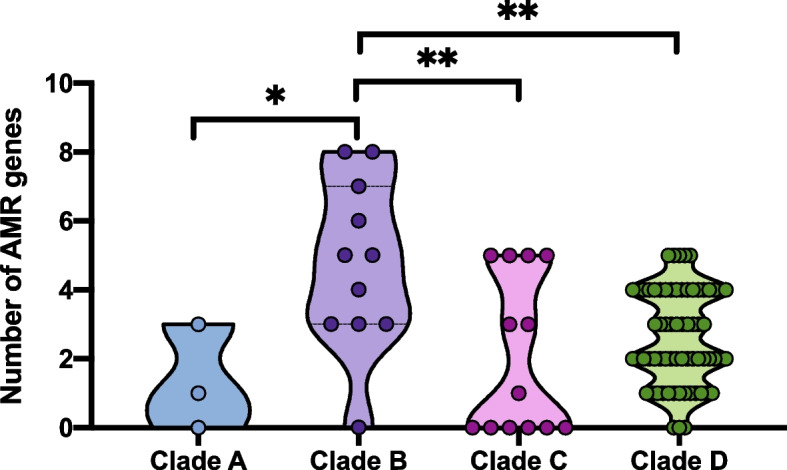
The number of AMR genes in four *M. caseolyticus* clades

Given the importance of antimicrobial resistance (AMR) in opportunistic pathogens, we compared the distributions of AMR genes in 94 M*. caseolyticus* strains from different clades (Fig. 2). A total of 24 AMR genes associated with 10 classes of antimicrobial agents were present in the isolates from four clades, among which 33% (1/3, clade A), 90% (9/10, clade B), 50% (7/14, clade C) and 46.3% (31/67, clade D) of isolates from the corresponding clades were identified as multi-drug resistance (Fig. 2). Notably, all the nine Chinese isolates contained *mecB* gene whiles HB024566, HB024567, and HB024568 carried multi-antimicrobial resistance genes including *ant(6)-Ia, drfG, isaE, inuB, qacZ, and tetL.* In addition, *tetL* was detected in all present isolates from Shanghai city, China, which may be associated with the horizontal plasmid transfer of rep22. The mobile genetic elements analysis showed that rep7a, rep22, and repUS56 were the top dominant plasmids in *M. caseolyticus* with the prevalence of 35%, 35%, and 34% in global clades, respectively (Fig. 2). Further statics indicated that the number of AMR genes from clade B (Chinese clade) was significantly higher than those from clade A, C and D (* *p* < 0.05, ** *p* < 0.01, Fig. 3).

### Phenotypical antimicrobial resistance of present strains

All nine *M. caseolyticus* isolates from present study were screened for phenotypic antimicrobial-resistant profiles. Specifically, all nine isolates were resistant to ampicillin, cefazolin, ceftazidime, lincomycin, piperacillin, penicillin, streptomycin, and tetracycline, whereas a majority were sensitive to amikacin, cefuroxime, and gentamicin, indicating a broad but complex multi-drug resistance in these *M. caseolyticus* strains isolated from the current retail meat in Shanghai, China (Table 2).

**Table 2:** Phenotypic antimicrobial susceptibility of nine *M. caseolyticus* isolates via disk diffusion test

Strains	AMP	AMK	CZO	CXM	CRO	CAZ	CFP	DOXY	ERY	GEM	KAN	LIN	MNO	PEN	PIP	STR	TET
HB024538	R	S	R	R	R	R	R	R	R	S	R	R	I	R	R	R	R
HB024539	R	I	R	R	I	R	I	R	R	S	R	R	R	R	R	R	R
HB024564	R	I	R	S	I	R	R	R	R	S	I	R	R	R	R	R	R
HB024565	R	R	R	R	I	R	R	R	R	I	R	R	R	R	R	R	R
HB024566	R	S	R	S	I	R	I	R	R	S	R	R	R	R	R	R	R
HB024567	R	S	R	S	I	R	R	R	R	S	R	R	R	R	R	R	R
HB024568	R	S	R	S	I	R	R	R	R	S	R	R	I	R	R	R	R
HB024569	R	R	R	I	R	R	R	I	R	R	I	R	R	R	R	R	R
HB024570	R	R	R	R	S	R	R	R	R	S	R	R	R	R	R	R	R

*AMP* Ampicillin, *AMK* Amikacin, *CZO* Cefazolin, *CXM* Cefuroxime, *CRO* Ceftriaxone, *CAZ* Ceftazidime, *CFP* Cefoperazone, *DOXY* Doxycycline, *ERY* Erythromycin, *GEM* Gentamicin, *KAN* Kanamycin, *LIN* Lincomycin, *MNO* Minocycline, *PEN* Penicillin, *PIP* Piperacillin, *STR* Streptomycin, *TET* Tetracycline. S stands for sensitive, I for intermediate, and R for resistant

### Distributions of virulence factors in global clades

**Fig. 4:**
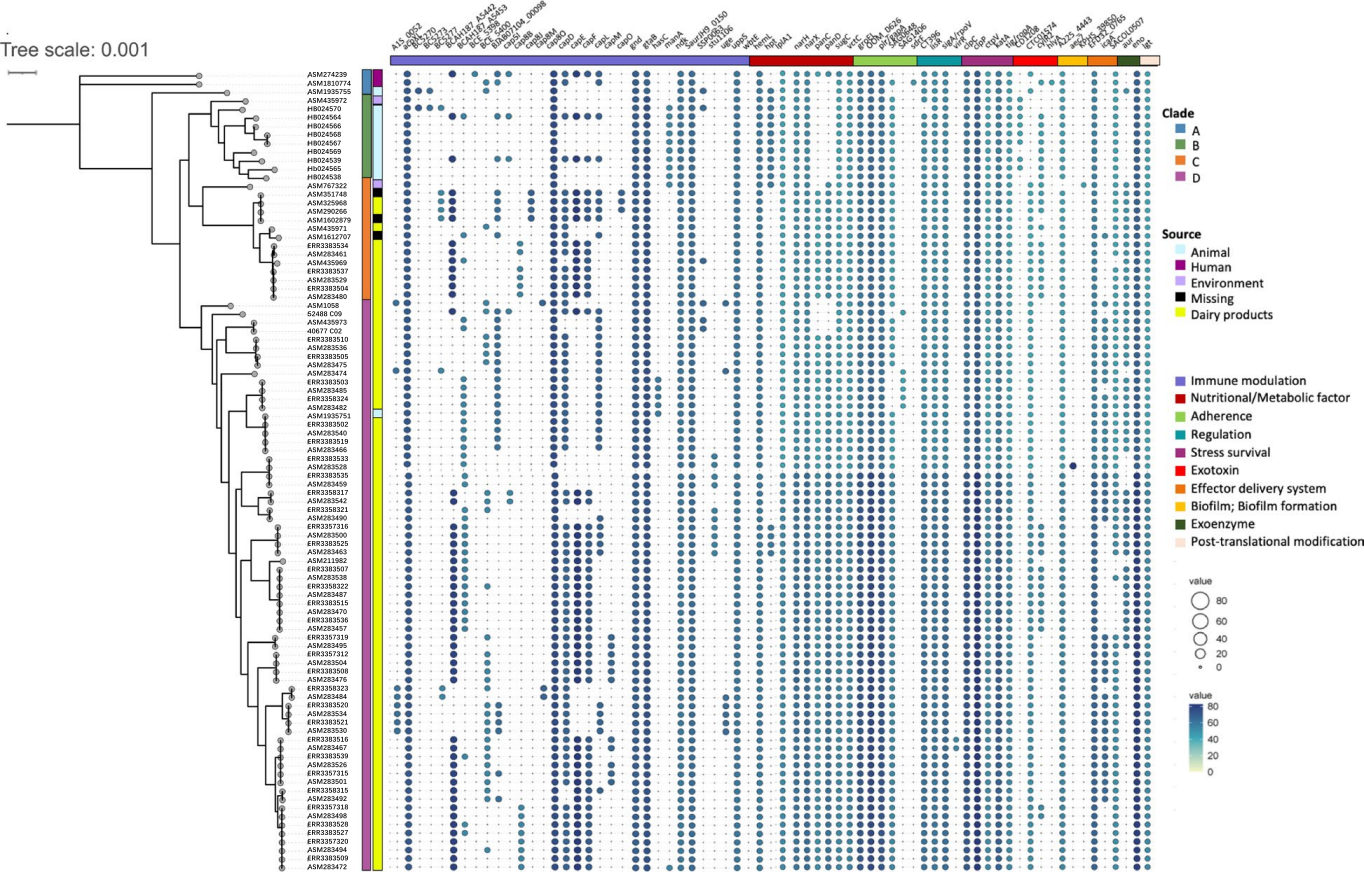
Distribution of virulence factors in global *M. caseolyticus* clades. The larger the diameter of the circle and the darker the color, the higher the confidence value. Squares are colored by feature class, representing the presence of the checked features

**Fig. 5:**
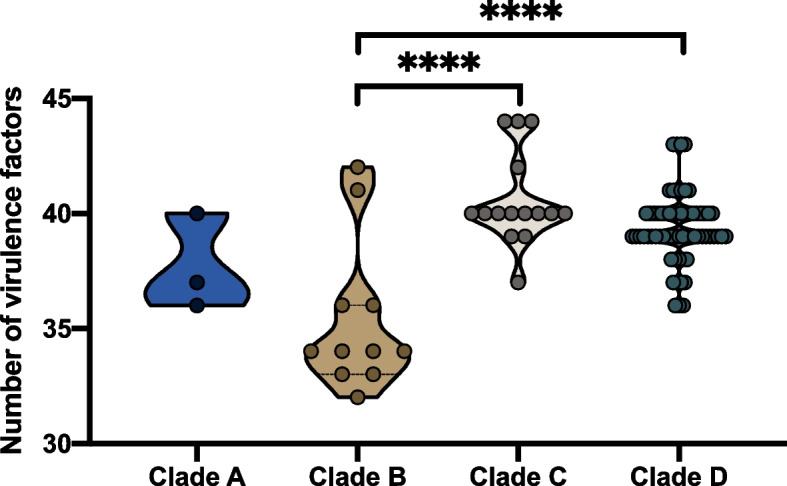
The number of virulence factors in four *M. caseolyticus* clades

Virulence factors were compared to determine the pathogenicity within the four clades (Fig. 4). Totally 69 virulence factors were assessed among 94 M*. caseolyticus* strains including the functional factors of adherence, biofilm formation, exotoxin, capsule, and others. Remarkably, the majority of Chinese clade B lacked the factor of CTC01574 (hemolysin) but contained CD1208 which were associated with hemolysin. Meanwhile, most clade B strains contained cytolysin-related genes, *e.g., hpt*, *manA*, however, lacked genes regulating capsule synthesis such as *capE*, *capF*, *capO*, and *capM* (capsular polysaccharide synthesis enzyme), indicating the weak ability of invasiveness. In clade D, the replacement and loss of functional genes were also observed that KPHS_39850 (protein disaggregation chaperone) was only present in ASM767322 while A225_4443 (*clpB* factor) were widely distributed in all global isolates. Notably, the *aur panC,* and *panD* genes were absent in Chinese isolates in clade B. To summarize, the number of virulence factors in clade B was significantly lower than that of clade C and D (**** *p* < 0.001, Fig. 5), which may be due to the deletion of corresponding proteins related to capsule synthesis, zinc metalloproteinase aureolysin, and pantothenic acid synthesis.

### Comparative morphological observation of *M. caseolyticus* and *S. aureus*

**Fig. 6:**
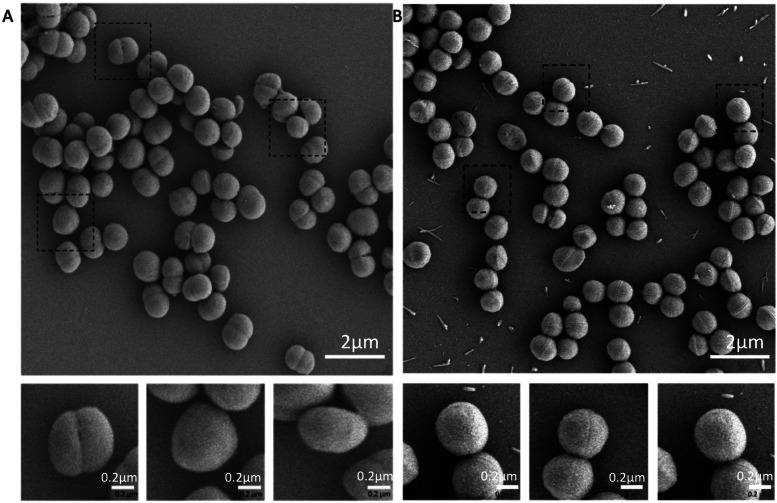
Scanning electron micrographs of **A** *M. caseolyticus* HB024567 and **B** *S.aureus* ATCC 25,923

**Fig. 7:**
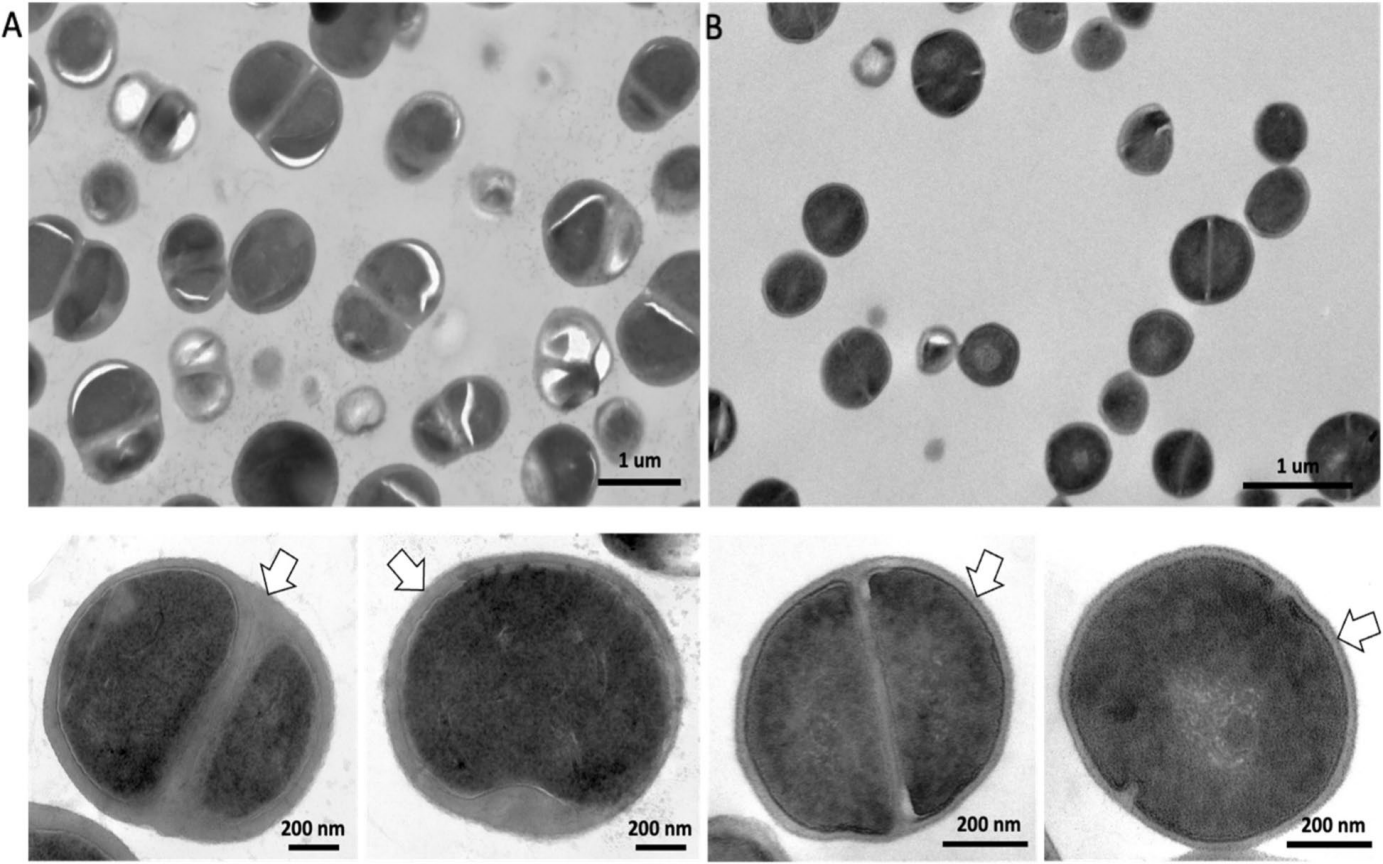
Transmission electron micrographs of **A** *M. caseolyticus* HB024567 and **B** *S.aureus* ATCC 25,923

To better understand the morphological differences between *M. caseolyticus* and its closest relative of *S. aureus*, the HB024567 strain from present study and *S. aureus* ATCC 25,923 were employed for SEM and TEM analysis. SEM results showed that *M. caseolyticus* was 1.1 ± 0.05 μm with a round shape and smooth surface, while *S. aureus* was 0.46 ± 0.01 μm which was smaller than the *M. caseolyticus* strain (Fig. 6). Then the TEM results demonstrated that *M. caseolyticus* had a broad and thick cell wall with a diameter of around 65 ± 5 nm, whereas the cell wall was much narrow and thin in *S. aureus* (21 ± 1 nm) (Fig. 7).

## Discussion

In China, *Macrococcus caseolyticus* has been isolated from many fermented foods, such as Chinese sausages and the specialty food "ChouGuiYu", a type of fermented mandarin fish [6]. Furthermore, *M. caseolyticus* can be isolated from human, animal, and raw meat during food processing and transportation [1, 16, 17], allowing it to be transmitted internationally via commercial shipments of agricultural products.

China is the largest import market and the 6^th^ largest export market of agricultural products. With these commercial shipments it was expected that *M. caseolyticus* in China would be greatly sharpened. To evaluate the hypothesis, *M. caseolyticus* were isolated from both beef (*n* = 7) and pork (*n* = 2) from Shanghai. Nine isolates presented a novel sequence type. In particular, six isolates were found to each carry > 3 novel alleles, indicating that they were genetically distinct from all existing isolates in pubMLST. Phylogenetic analysis also confirmed that nine Chinese *M. caseolyticus* isolates formed a distinct cluster of Clade B in the tree, which did not intermingle with isolates from other countries, with the exception of one isolate from the US (ASM435972).

Most *M. caseolyticus* isolates carried at least one of *mecB* and *mecD* elements (Fig. 2), both homologs of *mecA*, which induced resistance to methicillin [28]. A recent study also reported the presence of *mecB* in *S. aureus* strains, which could be horizontally transferred from *M. caseolyticus* [2]. All nine Chinese isolates carried the *mecB* gene. Furthermore, six Chinese isolates were multidrug-resistant, probably due to the acquisitions of MDR mobile genetic elements. For example, it was identified that *str* gene was carried by rep7a, *aadD* (aminoglycoside nucleotidyltransferase gene) and *tetL* (tetracycline efflux protein gene) genes were carried by rep22, among others. Intriguingly, the Chinese isolates in Clade B had the more AMR genes then other isolates. This may be associated with the higher usage of antimicrobial agents in Chinese veterinary [20].

Antimicrobial resistance was tested phenotypically and associated encoding genes identified in genome-based analysis of all nine *M. caseolyticus* strains. In silico detection showed that *M. caseolyticus* isolates harbored antimicrobial resistance genes supporting the results of the phenotypical resistance to methicillin, tetracycline, and streptomycin including the *mecB* gene (*n* = 9), *tetL* genes (*n* = 9), *srt* genes (*n* = 9), and other genes (Fig. 2 and Table 2).

Comparing the findings regarding the prevalence of antimicrobial resistant *M. caseolyticus* in this study with other studies. A regional-wide investigation on *M. caseolyticus* in England and Wales shows that all strains (*n* = 33) were determined as methicillin-resistant with both *mecB* and *mecD* detected in these diverse isolates [23]. A Chinese study describes the isolation of *M. caseolyticus* from broiler chickens containing genes that confer resistance to amikacin, penicillinG, piperacillin, kanamycin, chloramphenicin, and ceftriaxone, which is similar to our findings in the present study [20].

At the same time, all *M. caseolyticus* genomes were screened for virulence factors. The Chinese *M. caseolyticus* isolates were clustered together with ASM435972. Comparative genomic analysis revealed several functional gene substitutions and losses between ASM435972 and the other clade B isolates. Notably, genes related to capsule synthesis were absent in Chinese isolates, which have previously been reported as the dominant virulence factors in broiler disease [20]. The *panC* and *panD* genes with pantothenic acid synthesis functions were also lost in the present Chinese isolates. Such reduction in virulence factors in Chinese *M. caseolyticus* isolates indicated a weaker pathogenicity, which might be due to the isolated environment of retail raw meat, rather than human skin or nasal samples.

Scanning electron microscopy (SEM) and transmission electron microscopy (TEM) results showed that *M. caseolyticus* HB024567 possessed a diameter of 1.1 ± 0.05 μm with a cell wall of 65 ± 5 nm (Fig. 6 A & B), which showed larger values of diameter and cell wall compared to *S. aureus* ATCC 25,923. The genus *Macrococcus* was measured in previous studies with the diameters of *M.caseolyticus* ATCC13548 (1.1–2 μm), *M.equipercicus* ATCC51831 (1.3–2.3 μm), *M. bovicus* ATCC51825 (1.2–2.1 μm), *M.carouselicus* ATCC5128 (1.4–2.5 μm), *M. brunensis* CCM4811 (0.89 -1.2 μm), *M. hajekii* CCM4809 (0.89 μm), *M. lamae* CCM4815 (0.74 -0.92 μm), and *M. canis* KM45013 (0.8 μm) [4, 9, 18, 24]. SEM results showed that *M. caseolyticus* had a smooth surface, while it was rough in *M. bovicus*, *M. equipercicus* and *M. carouselicus*, with spiny protrusions observed in *M. equipercicus* stains [18].

In conclusion, comparative genomic analyses were enrolled for the construction of population structure as well as for the prediction of antimicrobial resistance and virulence factors in 94 M*. caseolyticus* strains collected from humans, animals, meat, and dairy products. Four clades were identified globally that Chinese isolates clustered together as clade B, carrying significantly more AMR genes (*p* < 0.05) and less virulence factors (*p* < 0.001). MLST typing and phylogenetic trees indicated a potential local evolution of *M. caseolyticus* in China. The comparative electron microscope demonstrated the morphological variation between *M. caseolyticus* and *S. aureus,* showing that *M. caseolyticus* has a larger diameter and thicker cell walls. Together, the present study provided a phylogenetic and genotypic comparison of the global *M. caseolyticus* stains. Our results suggested that both human and animal reservoirs could contribute to contamination in food products and such products might serve as a vector for *M. caseolyticus* habitats in foodborne microorganisms.

## Supplementary Information

**Additional file 1:** **Supply Table 1.** Isolation and identification of nine *M. caseolyticus* isolates from this study by fIDBAC (http://fbac.dmicrobe.cn). **Supply Table 2.** Description of the genomes used in this study. A total of 85 global strains were downloaded from GenBank including 34 sets of filtered SRA genomes and 51 already assemblies. **Supply Table 3.** Description of the 24 antimicrobial resistance genes used in this study. **Supply Table 4.** Multi-locus sequence typing (MLST) identified in additional 85 global *M. caseolyticus* isolates. 

## Data Availability

The raw reads from this study are submitted to the China National GenBank under project accession ID CNP0002826.

## References

[1] Acheampong OD, Enyetornye B, Osei D (2021). Polymicrobial necrotizing fasciitis in a dog: the involvement of *Macrococcus caseolyticus*, *Proteus mirabilis*, and *Escherichia coli*. Case Rep Vet Med.

[2] Becker K, van Alen S, Idelevich EA, Schleimer N, Seggewiß J, Mellmann A, Kaspar U, Peters G (2018). Plasmid-encoded transferable *mecB*-mediated methicillin resistance in *Staphylococcus aureus*. Emerg Infect Dis.

[3] Bortolaia V, Kaas RS, Ruppe E, Roberts MC, Schwarz S, Cattoir V, Philippon A, Allesoe RL, Rebelo AR, Florensa AF, Fagelhauer L, Chakraborty T, Neumann B, Werner G, Bender JK, Stingl K, Nguyen M, Coppens J, Xavier BB, Malhotra-Kumar S, Aarestrup FM (2020). ResFinder 4.0 for predictions of phenotypes from genotypes. J Antimicrob Chemother.

[4] Brawand SG, Cotting K, Gómez-Sanz E, Collaud A, Thomann A, Brodard I, Rodriguez-Campos S, Strauss C, Perreten V (2017). *Macrococcus canis* sp. nov., a skin bacterium associated with infections in dogs. Int J Syst Evol Microbiol.

[5] Cotting K, Strauss C, Rodriguez-Campos S, Rostaher A, Fischer NM, Roosje PJ, Favrot C, Perreten V (2017). *Macrococcus canis* and *M. caseolyticus* in dogs: occurrence, genetic diversity and antibiotic resistance. Vet Dermatol.

[6] Dai Z, Li Y, Wu J, Zhao Q (2013). Diversity of lactic acid bacteria during fermentation of a traditional Chinese fish product, Chouguiyu (stinky mandarin fish). J Food Sci.

[7] de la Fuente R, Suarez G, Ruiz Santa Quiteria JA, Meugnier H, Bes M, Freney J, Fleurette J (1992). Identification of coagulase negative staphylococci isolated from lambs as *Staphylococcus caseolyticus*. Comp Immunol Microbiol Infect Dis.

[8] Didelot X, Croucher NJ, Bentley SD, Harris SR, Wilson DJ (2018). Bayesian inference of ancestral dates on bacterial phylogenetic trees. Nucleic Acids Res.

[9] Evans AC. The bacteria of milk freshly drawn from normal udders. J Infect Dis. 1916;437–476. https://www.semanticscholar.org/paper/The-Bacteria-of-Milk-Freshly-Drawn-from-Normal-Evans/42dc33dd2ae10afde83468d494e04201016d5e49

[10] Fuka MM, Wallisch S, Engel M, Welzl G, Havranek J, Schloter M (2013). Dynamics of bacterial communities during the ripening process of different Croatian cheese types derived from raw ewe's milk cheeses. PLoS ONE.

[11] García-Álvarez L, Holden MT, Lindsay H, Webb CR, Brown DF, Curran MD, Walpole E, Brooks K, Pickard DJ, Teale C, Parkhill J, Bentley SD, Edwards GF, Girvan EK, Kearns AM, Pichon B, Hill RL, Larsen AR, Skov RL, Peacock SJ, Holmes MA (2011). Meticillin-resistant *Staphylococcus aureus* with a novel *mecA* homologue in human and bovine populations in the UK and Denmark: a descriptive study. Lancet Infect Dis.

[12] Gómez-Sanz E, Schwendener S, Thomann A, Gobeli Brawand S, Perreten V (2015). First staphylococcal cassette chromosome mec containing a *mecB*-carrying gene complex independent of transposon Tn6045 in a *Macrococcus canis* isolate from a canine infection. Antimicrob Agents Chemother.

[13] Gurevich A, Saveliev V, Vyahhi N, Tesler G (2013). QUAST: quality assessment tool for genome assemblies. Bioinformatics (Oxford, England).

[14] Johansson M, Bortolaia V, Tansirichaiya S, Aarestrup FM, Roberts AP, Petersen TN (2021). Detection of mobile genetic elements associated with antibiotic resistance in *Salmonella enterica* using a newly developed web tool: MobileElementFinder. J Antimicrob Chemother.

[15] Joishy TK, Dehingia M, Khan MR (2019). Bacterial diversity and metabolite profiles of curd prepared by natural fermentation of raw milk and back sloping of boiled milk. World J Microbiol Biotechnol.

[16] Jost G, Schwendener S, Liassine N, Perreten V (2021). Methicillin-resistant *Macrococcus canis* in a human wound. Infect Genet Evol.

[17] Keller JE, Schwendener S, Neuenschwander J, Overesch G, Perreten V (2022). Prevalence and characterization of methicillin-resistant *Macrococcus* spp. in food producing animals and meat in Switzerland in 2019. Prävalenz und Charakterisierung von Methicillin-resistenten *Macrococcus* spp. bei Nutztieren und Fleisch in der Schweiz im Jahr 2019. Schweiz Arch Tierheilkd.

[18] Kloos WE, Ballard DN, George CG, Webster JA, Hubner RJ, Ludwig W, Schleifer KH, Fiedler F, Schubert K (1998). Delimiting the genus *Staphylococcus* through description of *Macrococcus caseolyticus* gen. nov., comb. nov. and *Macrococcus equipercicus* sp. nov., and *Macrococcus bovicus* sp. no. and *Macrococcus carouselicus* sp. nov. Int J Syst Bacteriol.

[19] Letunic I, Bork P (2019). Interactive Tree Of Life (iTOL) v4: recent updates and new developments. Nucleic Acids Res.

[20] Li G, Du X, Zhou D, Li C, Huang L, Zheng Q, Cheng Z (2018). Emergence of pathogenic and multiple-antibiotic-resistant *Macrococcus caseolyticus* in commercial broiler chickens. Transbound Emerg Dis.

[21] Li H, Tang T, Stegger M, Dalsgaard A, Liu T, Leisner JJ (2021). Characterization of antimicrobial-resistant *Staphylococcus aureus* from retail foods in Beijing, China. Food Microbiol.

[22] Liu B, Zheng D, Jin Q, Chen L, Yang J (2019). VFDB 2019: a comparative pathogenomic platform with an interactive web interface. Nucleic Acids Res.

[23] MacFadyen AC, Fisher EA, Costa B, Cullen C, Paterson GK (2018). Genome analysis of methicillin resistance in *Macrococcus caseolyticus* from dairy cattle in England and Wales. Microbial genomics.

[24] Mannerová S, Pantůček R, Doškař J, Švec P, Snauwaert C, Vancanneyt M, Swings J, Sedláček I (2003). *Macrococcus brunensis* sp. nov., *Macrococcus hajekii* sp. nov. and *Macrococcus lamae* sp. nov., from the skin of llamas. Int J Syst Evol Microbiol.

[25] Mašlaňová I, Wertheimer Z, Sedláček I, Švec P, Indráková A, Kovařovic V, Schumann P, Spröer C, Králová S, Šedo O, Krištofová L, Vrbovská V, Füzik T, Petráš P, Zdráhal Z, Ružičková V, Doškař J, Pantuček R (2018). Description and comparative genomics of *Macrococcus caseolyticus* subsp. *hominis* subsp. nov., *Macrococcus goetzii* sp. nov., *Macrococcus epidermidis* sp. nov., and *Macrococcus bohemicus* sp. nov., novel Macrococci from human clinical material with virulence potential and suspected uptake of foreign DNA by natural transformation. Front Microbiol.

[26] Rantsiou K, Urso R, Iacumin L, Cantoni C, Cattaneo P, Comi G, Cocolin L (2005). Culture-dependent and -independent methods to investigate the microbial ecology of Italian fermented sausages. Appl Environ Microbiol.

[27] Sagulenko P, Puller V, Neher RA (2018). Treetime: maximum-likelihood phylodynamic analysis. Virus Evol.

[28] Schwendener S, Cotting K, Perreten V (2017). Novel methicillin resistance gene *mecD* in clinical *Macrococcus caseolyticus* strains from bovine and canine sources. Sci Rep.

[29] Tsubakishita S, Kuwahara-Arai K, Baba T, Hiramatsu K (2010). Staphylococcal cassette chromosome *mec*-like element in *Macrococcus caseolyticus*. Antimicrob Agents Chemother.

[30] Ubukata K, Nonoguchi R, Matsuhashi M, Konno M (1989). Expression and inducibility in *Staphylococcus aureus* of the mecA gene, which encodes a methicillin-resistant *S. aureus*-specific penicillin-binding protein. J Bacteriol.

[31] Zhou Z, Alikhan NF, Mohamed K, Fan Y, Achtman M, Agama Study Group (2020). The EnteroBase user's guide, with case studies on *Salmonella* transmissions, *Yersinia pestis* phylogeny, and *Escherichia* core genomic diversity. Genome Res.

[32] Zhou Z, Alikhan NF, Sergeant MJ, Luhmann N, Vaz C, Francisco AP, Carriço JA, Achtman M (2018). GrapeTree: visualization of core genomic relationships among 100,000 bacterial pathogens. Genome Res.

