# Supplementary material for: Characteristics of population structure, antimicrobial resistance, virulence factors, and morphology of methicillin-resistant Macrococcus caseolyticus in global clades
Source: BMC Microbiol. 2022 Nov 5;22:266. doi: 10.1186/s12866-022-02679-8 (PMC9636676; doi:10.1186/s12866-022-02679-8)
Supplement: Supplementary file 1 — Additional file 1: Supply Table 1. Isolation and identification of nine M. caseolyticus isolates from this study by fIDBAC (http://fbac.dmicrobe.cn). Supply Table 2. Description of the genomes used in this study. A total of 85 global strains were downloaded from GenBank including 34 sets of filtered SRA genomes and 51 already assemblies. Supply Table 3. Description of the 24 antimicrobial resistance genes used in this study. Supply Table 4. Multi-locus sequence typing (MLST) identified in additional 85 global M. caseolyticus isolates. [file 12866_2022_2679_MOESM1_ESM.docx]

**Supply materials**

**Supply table 1. Isolation and identification of nine *M. caseolyticus* isolates from this study by fIDBAC (**[**http://fbac.dmicrobe.cn**](http://fbac.dmicrobe.cn)**).**

**Supply table 2. Description of the genomes used in this study. A total of 85 global strains were downloaded from GenBank including 34 sets of filtered SRA genomes and 51 already assemblies.**

**Supply table 3. Description of the 24 antimicrobial resistance genes used in this study.**

**Supply table 4. Multi-locus sequence typing (MLST) identified in additional 85 global *M. caseolyticus* isolates.**

**Supply table 1. Isolation and Identification of nine *M. caseolyticus* isolates from this study by fIDBAC (**[**http://fbac.dmicrobe.cn**](http://fbac.dmicrobe.cn)**).**

| **Strain names** | **Source** | **Time** | **Location** | **ANI vs Type material Genome** | **Identity** |
| --- | --- | --- | --- | --- | --- |
| HB024538.result | Beef | 2021/9/7 | Shuicheng Road and Honggu Road in Changning District, Shanghai | Macrococcus_caseolyticus.CCM-3540.PZJF01 | 98.55 |
|  |  |  |  | Macrococcus_caseolyticus.CCM-7927.MJBJ02 | 96.96 |
|  |  |  |  | Macrococcus_caseolyticus.DSM-20597.PPRM01 | 98.55 |
| HB024539.result | Beef | 2021/9/7 | Shuicheng Road and Honggu Road in Changning District, Shanghai | Macrococcus_caseolyticus.CCM-3540.PZJF01 | 98.52 |
|  |  |  |  | Macrococcus_caseolyticus.CCM-7927.MJBJ02 | 96.95 |
|  |  |  |  | Macrococcus_caseolyticus.DSM-20597.PPRM01 | 98.51 |
| HB024564.result | Beef | 2021/8/30 | Yuyao Road, Jing'an District, Shanghai | Macrococcus_caseolyticus.CCM-3540.PZJF01 | 98.5 |
|  |  |  |  | Macrococcus_caseolyticus.CCM-7927.MJBJ02 | 96.9 |
|  |  |  |  | Macrococcus_caseolyticus.DSM-20597.PPRM01 | 98.5 |
| HB024565.result | Beef | 2021/8/30 | Yuyao Road, Jing'an District, Shanghai | Macrococcus_caseolyticus.CCM-3540.PZJF01 | 98.6 |
|  |  |  |  | Macrococcus_caseolyticus.CCM-7927.MJBJ02 | 96.99 |
|  |  |  |  | Macrococcus_caseolyticus.DSM-20597.PPRM01 | 98.58 |
| HB024566.result | Beef | 2021/8/30 | Yuyao Road, Jing'an District, Shanghai | Macrococcus_caseolyticus.CCM-3540.PZJF01 | 98.52 |
|  |  |  |  | Macrococcus_caseolyticus.CCM-7927.MJBJ02 | 96.93 |
|  |  |  |  | Macrococcus_caseolyticus.DSM-20597.PPRM01 | 98.52 |
| HB024567.result | Beef | 2021/8/30 | Yuyao Road, Jing'an District, Shanghai | Macrococcus_caseolyticus.CCM-3540.PZJF01 | 98.52 |
|  |  |  |  | Macrococcus_caseolyticus.CCM-7927.MJBJ02 | 96.95 |
|  |  |  |  | Macrococcus_caseolyticus.DSM-20597.PPRM01 | 98.52 |
| HB024568.result | Beef | 2021/8/30 | Yuyao Road, Jing'an District, Shanghai | Macrococcus_caseolyticus.CCM-3540.PZJF01 | 98.51 |
|  |  |  |  | Macrococcus_caseolyticus.CCM-7927.MJBJ02 | 96.95 |
|  |  |  |  | Macrococcus_caseolyticus.DSM-20597.PPRM01 | 98.51 |
| HB024569.result | Pork | 2021/8/31 | Fuxin Road, Yangpu District, Shanghai | Macrococcus_caseolyticus.CCM-3540.PZJF01 | 98.54 |
|  |  |  |  | Macrococcus_caseolyticus.CCM-7927.MJBJ02 | 96.93 |
|  |  |  |  | Macrococcus_caseolyticus.DSM-20597.PPRM01 | 98.49 |
| HB024570.result | Pork | 2021/9/8 | Zhangyang Road, Pudong New Area, Shanghai | Macrococcus_caseolyticus.CCM-3540.PZJF01 | 98.53 |
|  |  |  |  | Macrococcus_caseolyticus.CCM-7927.MJBJ02 | 96.9 |
|  |  |  |  | Macrococcus_caseolyticus.DSM-20597.PPRM01 | 98.52 |

**Supply table 2. Description of the genomes used in this study. A total of 85 global strains were downloaded from GenBank including 34 sets of filtered SRA genomes and 51 already assemblies.**

| **Selected** | **index** | **ID** | **country** | **year** |
| --- | --- | --- | --- | --- |
| SRA genomes | 1 | ERR3357312 | UK | 2019 |
| SRA genomes | 2 | ERR3357315 | UK | 2019 |
| SRA genomes | 3 | ERR3357316 | UK | 2019 |
| SRA genomes | 4 | ERR3357318 | UK | 2019 |
| SRA genomes | 5 | ERR3357319 | UK | 2019 |
| SRA genomes | 6 | ERR3357320 | UK | 2019 |
| SRA genomes | 7 | ERR3358315 | UK | 2019 |
| SRA genomes | 8 | ERR3358317 | UK | 2019 |
| SRA genomes | 9 | ERR3358321 | UK | 2019 |
| SRA genomes | 10 | ERR3358322 | UK | 2019 |
| SRA genomes | 11 | ERR3358323 | UK | 2019 |
| SRA genomes | 12 | ERR3358324 | UK | 2019 |
| SRA genomes | 13 | ERR3383502 | UK | 2019 |
| SRA genomes | 14 | ERR3383503 | UK | 2019 |
| SRA genomes | 15 | ERR3383504 | UK | 2019 |
| SRA genomes | 16 | ERR3383505 | UK | 2019 |
| SRA genomes | 17 | ERR3383507 | UK | 2019 |
| SRA genomes | 18 | ERR3383508 | UK | 2019 |
| SRA genomes | 19 | ERR3383509 | UK | 2019 |
| SRA genomes | 20 | ERR3383510 | UK | 2019 |
| SRA genomes | 21 | ERR3383515 | UK | 2019 |
| SRA genomes | 22 | ERR3383516 | UK | 2019 |
| SRA genomes | 23 | ERR3383519 | UK | 2019 |
| SRA genomes | 24 | ERR3383520 | UK | 2019 |
| SRA genomes | 25 | ERR3383521 | UK | 2019 |
| SRA genomes | 26 | ERR3383525 | UK | 2019 |
| SRA genomes | 27 | ERR3383527 | UK | 2019 |
| SRA genomes | 28 | ERR3383528 | UK | 2019 |
| SRA genomes | 29 | ERR3383533 | UK | 2019 |
| SRA genomes | 30 | ERR3383534 | UK | 2019 |
| SRA genomes | 31 | ERR3383535 | UK | 2019 |
| SRA genomes | 32 | ERR3383536 | UK | 2019 |
| SRA genomes | 33 | ERR3383537 | UK | 2019 |
| SRA genomes | 34 | ERR3383539 | UK | 2019 |
| Already assemblies | 35 | ASM1058 | Japan | 2009 |
| Already assemblies | 36 | ASM211982 | Switzerland | 2015 |
| Already assemblies | 37 | ASM274239 | Czech Republic | 2003 |
| Already assemblies | 38 | ASM283457 | UK | 2016 |
| Already assemblies | 39 | ASM283459 | UK | 2016 |
| Already assemblies | 40 | ASM283461 | UK | 2016 |
| Already assemblies | 41 | ASM283463 | UK | 2016 |
| Already assemblies | 42 | ASM283466 | UK | 2016 |
| Already assemblies | 43 | ASM283467 | UK | 2016 |
| Already assemblies | 44 | ASM283470 | UK | 2016 |
| Already assemblies | 45 | ASM283472 | UK | 2016 |
| Already assemblies | 46 | ASM283474 | UK | 2015 |
| Already assemblies | 47 | ASM283475 | UK | 2015 |
| Already assemblies | 48 | ASM283476 | UK | 2016 |
| Already assemblies | 49 | ASM283480 | UK | 2015 |
| Already assemblies | 50 | ASM283482 | UK | 2015 |
| Already assemblies | 51 | ASM283484 | UK | 2015 |
| Already assemblies | 52 | ASM283485 | UK | 2015 |
| Already assemblies | 53 | ASM283487 | UK | 2015 |
| Already assemblies | 54 | ASM283490 | UK | 2015 |
| Already assemblies | 55 | ASM283492 | UK | 2015 |
| Already assemblies | 56 | ASM283494 | UK | 2015 |
| Already assemblies | 57 | ASM283495 | UK | 2015 |
| Already assemblies | 58 | ASM283498 | UK | 2015 |
| Already assemblies | 59 | ASM283500 | UK | 2015 |
| Already assemblies | 60 | ASM283501 | UK | 2015 |
| Already assemblies | 61 | ASM283504 | UK | 2015 |
| Already assemblies | 62 | ASM283526 | UK | 2016 |
| Already assemblies | 63 | ASM283528 | UK | 2016 |
| Already assemblies | 64 | ASM283529 | UK | 2016 |
| Already assemblies | 65 | ASM283530 | UK | 2016 |
| Already assemblies | 66 | ASM283534 | UK | 2016 |
| Already assemblies | 67 | ASM283536 | UK | 2016 |
| Already assemblies | 68 | ASM283538 | UK | 2016 |
| Already assemblies | 69 | ASM283540 | UK | 2015 |
| Already assemblies | 70 | ASM283542 | UK | 2015 |
| Already assemblies | 71 | ASM290266 | Germany | 2018 |
| Already assemblies | 72 | ASM325968 | USA | 2018 |
| Already assemblies | 73 | ASM351748 | Australia | 2018 |
| Already assemblies | 74 | ASM435969 | Ireland | 2017 |
| Already assemblies | 75 | ASM435971 | Ireland | 2017 |
| Already assemblies | 76 | ASM435972 | USA | 2018 |
| Already assemblies | 77 | ASM435973 | Ireland | 2017 |
| Already assemblies | 78 | ASM767322 | USA | 2020 |
| Already assemblies | 79 | ASM1602879 | USA | 2013 |
| Already assemblies | 80 | ASM1612707 | Germany | 2013 |
| Already assemblies | 81 | ASM1810774 | Sudan | 2016 |
| Already assemblies | 82 | ASM1935751 | Switzerland | 2019 |
| Already assemblies | 83 | ASM1935755 | Switzerland | 2019 |
| Already assemblies | 84 | 52488_C09 | UK | 2018 |
| Already assemblies | 85 | 40677_C02 | UK | 2018 |
| This study | 86 | HB024538 | China | 2021 |
| This study | 87 | HB024539 | China | 2021 |
| This study | 88 | HB024564 | China | 2021 |
| This study | 89 | HB024565 | China | 2021 |
| This study | 90 | HB024566 | China | 2021 |
| This study | 91 | HB024567 | China | 2021 |
| This study | 92 | HB024568 | China | 2021 |
| This study | 93 | HB024569 | China | 2021 |
| This study | 94 | HB024570 | China | 2021 |

**Supply table 3. Description of the antimicrobial resistance genes detected in *M. caseolyticus* isolates.**

| **Antimicrobial resistance gene** | **Function** | **Link** |
| --- | --- | --- |
| *mecD* | methicillin resistant PBP2 gene，mediates penam resistance | <https://card.mcmaster.ca/ontology/41338> |
| *mecB* | methicillin resistant PBP2 gene，mediates penam resistance | <https://card.mcmaster.ca/ontology/40024> |
| *aadD ant(4',4'')* | aminoglycoside nucleotidyltransferase gene，mediates aminoglycoside antibiotic resistance | <https://card.mcmaster.ca/ontology/39023> |
| *aph(2'')-Ia* | aminoglycoside nucleotidyltransferase gene，mediates aminoglycoside antibiotic resistance | <https://card.mcmaster.ca/ontology/38997> |
| *aph(2'')-Ic* | aminoglycoside nucleotidyltransferase gene，mediates aminoglycoside antibiotic resistance | <https://card.mcmaster.ca/ontology/39036> |
| *aph(3')-III* | aminoglycoside nucleotidyltransferase gene，mediates aminoglycoside antibiotic resistance | <https://card.mcmaster.ca/ontology/39047> |
| *ant(6)-Ia* | aminoglycoside nucleotidyltransferase gene，mediates aminoglycoside antibiotic resistance | <https://card.mcmaster.ca/ontology/39026> |
| *ant(9)-Ia* | aminoglycoside nucleotidyltransferase gene，mediates aminoglycoside antibiotic resistance | <https://card.mcmaster.ca/ontology/39030> |
| *aac(6')-aph(2'')* | aminoglycoside nucleotidyltransferase gene，mediates aminoglycoside antibiotic resistance | <https://card.mcmaster.ca/ontology/38997> |
| *dfrG* | dihydrofolate reductase gene，mediates diaminopyrimidine antibiotic resistance | <https://card.mcmaster.ca/ontology/39302> |
| *dfrK* | dihydrofolate reductase gene，mediates diaminopyrimidine antibiotic resistance | <https://card.mcmaster.ca/ontology/39303> |
| *lsaE* | lsa-type ABC-F protein gene，mediates lincosamide antibiotic, pleuromutilin antibiotic, streptogramin antibiotic resistance | <https://card.mcmaster.ca/ontology/39790> |
| *lun(A)* | lincosamide nucleotidyltransferase gene，mediates lincosamide antibiotic resistance | <https://card.mcmaster.ca/ontology/36360> |
| *lun(B)* | lincosamide nucleotidyltransferase gene，mediates lincosamide antibiotic resistance | <https://card.mcmaster.ca/ontology/36360> |
| *lun(G)* | lincosamide nucleotidyltransferase gene，mediates lincosamide antibiotic resistance | <https://card.mcmaster.ca/ontology/36360> |
| *qacG* | small multidrug resistance efflux pump gene，mediates fluoroquinolone antibiotic resistance | <https://card.mcmaster.ca/ontology/45484> |
| *qacZ* | small multidrug resistance efflux pump gene，mediates fluoroquinolone antibiotic resistance | <https://card.mcmaster.ca/ontology/39480> |
| *str* | streptothricin acetyltransferase gene，mediates nucleoside antibiotic resistance | <https://card.mcmaster.ca/ontology/35931> |
| *tet(K)* | tetracycline efflux protein gene，mediates tetracycline antibiotic resistance | <https://card.mcmaster.ca/ontology/36317> |
| *tet(L)* | tetracycline efflux protein gene，mediates tetracycline antibiotic resistance | <https://card.mcmaster.ca/ontology/36318> |
| *tet(M)* | tetracycline efflux protein gene，mediates tetracycline antibiotic resistance | <https://card.mcmaster.ca/ontology/36325> |
| *tet(S)* | tetracycline efflux protein gene，mediates tetracycline antibiotic resistance | <https://card.mcmaster.ca/ontology/36331> |
| *fusC* | 2-domain zinc-binding protein gene，mediates fusidane antibiotic resistance | <https://card.mcmaster.ca/ontology/40388> |
| *ermB* | 23S ribosomal RNA methyltransferase gene，mediates streptogramin antibiotic, macrolide antibiotic, lincosamide antibiotic, streptogramin A antibiotic, streptogramin B antibiotic resistance | <https://card.mcmaster.ca/ontology/36514> |

**Supply table 4. Multi-locus sequence typing (MLST) identified in additional 85 global *M. caseolyticus* isolates.**

| **Strains** | **MLST#** | **ack** | **cpn60** | **fdh** | **pta** | **purA** | **sar** | **tuf** |
| --- | --- | --- | --- | --- | --- | --- | --- | --- |
| ERR3357312 | 40 | 6 | 11 | 11 | 2 | 12 | 3 | 2 |
| ERR3357315 | 21 | 6 | 4 | 11 | 2 | 5 | 3 | 2 |
| ERR3357316 | 41 | 15 | 3 | 11 | 2 | 5 | 3 | 3 |
| ERR3357318 | 26 | 6 | 4 | 5 | 2 | 5 | 3 | 2 |
| ERR3357319 | 42 | 15 | 13 | 13 | 2 | 12 | 6 | 2 |
| ERR3357320 | 26 | 6 | 4 | 5 | 2 | 5 | 3 | 2 |
| ERR3358315 | 43 | 6 | 4 | 15 | 2 | 5 | 3 | 3 |
| ERR3358317 | 44 | 6 | 3 | 11 | 2 | 5 | 3 | 3 |
| ERR3358321 | 45 | 6 | 3 | 5 | 2 | 5 | 6 | 3 |
| ERR3358322 | 5 | 5 | 3 | 5 | 2 | 5 | 3 | 2 |
| ERR3358323 | 6 | 4 | 4 | 5 | 2 | 5 | 3 | 2 |
| ERR3358324 | 46 | 5 | 9 | 5 | 7 | 10 | 6 | 2 |
| ERR3383502 | 47 | 6 | 3 | 5 | 7 | 5 | 6 | 2 |
| ERR3383503 | 46 | 5 | 9 | 5 | 7 | 10 | 6 | 2 |
| ERR3383504 | 48 | 6 | 3 | 4 | 3 | 6 | 3 | 2 |
| ERR3383505 | 49 | 6 | 8 | 5 | 7 | 5 | 14 | 2 |
| ERR3383507 | 5 | 5 | 3 | 5 | 2 | 5 | 3 | 2 |
| ERR3383508 | 40 | 6 | 11 | 11 | 2 | 12 | 3 | 2 |
| ERR3383509 | 26 | 6 | 4 | 5 | 2 | 5 | 3 | 2 |
| ERR3383510 | 49 | 6 | 8 | 5 | 7 | 5 | 14 | 2 |
| 40677_C02 | 28 | 11 | 3 | 5 | 7 | 8 | 3 | 3 |
| 52488_C09 | #10/#15/#16/#21/#24/  #26/#33/#34/40/43  /44/45/51/60/62 | 6 | 6 | 8 | 2 | 8 | 1 | ND |
| ASM1058v1 | #31 | 3 | 3 | 15*/8* | 7 | 3 | 11 | 2 |
| ASM211982v1 | 5 | 5 | 3 | 5 | 2 | 5 | 3 | 2 |
| ASM274239v2 | #10/#61/#67 | 13* | 13* | 7* | 4* | 2/14 | 17* | 12 |
| ASM283457v1 | 5 | 5 | 3 | 5 | 2 | 5 | 3 | 2 |
| ERR3383515 | 5 | 5 | 3 | 5 | 2 | 5 | 3 | 2 |
| ERR3383516 | 51 | 6 | 4 | 11 | 2 | 5 | 3 | 9 |
| ERR3383519 | 47 | 6 | 3 | 5 | 7 | 5 | 6 | 2 |
| ERR3383520 | 6 | 4 | 4 | 5 | 2 | 5 | 3 | 2 |
| ERR3383521 | 6 | 4 | 4 | 5 | 2 | 5 | 3 | 2 |
| ERR3383525 | #41/#65 | 15 | 3 | 11 | 2 | 5 | 3 | 3 |
| ERR3383527 | 26 | 6 | 4 | 5 | 2 | 5 | 3 | 2 |
| ERR3383528 | 26 | 6 | 4 | 5 | 2 | 5 | 3 | 2 |
| ERR3383533 | 26 | 6 | 4 | 5 | 2 | 5 | 3 | 2 |
| ERR3383534 | 48 | 6 | 3 | 4 | 3 | 6 | 3 | 2 |
| ERR3383535 | 52 | 16 | 3 | 5 | 7 | 17 | 6 | 2 |
| ERR3383536 | 5 | 5 | 3 | 5 | 2 | 5 | 3 | 2 |
| ERR3383537 | 48 | 6 | 3 | 4 | 3 | 6 | 3 | 2 |
| ERR3383539 | 21 | 6 | 4 | 11 | 2 | 5 | 3 | 2 |
| ASM283459v1 | 52 | 16 | 3 | 5 | 7 | 17 | 6 | 2 |
| ASM283461v1 | 48 | 6 | 3 | 4 | 3 | 6 | 3 | 2 |
| ASM283463v1 | 41 | 15 | 3 | 11 | 2 | 5 | 3 | 3 |
| ASM283466v1 | 47 | 6 | 3 | 5 | 7 | 5 | 6 | 2 |
| ASM283467v1 | 51 | 6 | 4 | 11 | 2 | 5 | 3 | 9 |
| ASM283470v1 | 5 | 5 | 3 | 5 | 2 | 5 | 3 | 2 |
| ASM283472v1 | 26 | 6 | 4 | 5 | 2 | 5 | 3 | 2 |
| ASM283474v1 | 50 | 16 | 3 | 16 | 2 | 16 | 3 | 2 |
| ASM283475v1 | 49 | 6 | 8 | 5 | 7 | 5 | 14 | 2 |
| ASM283476v1 | 40 | 6 | 11 | 11 | 2 | 12 | 3 | 2 |
| ASM283480v1 | 48 | 6 | 3 | 4 | 3 | 6 | 3 | 2 |
| ASM283482v1 | 46 | 5 | 9 | 5 | 7 | 10 | 6 | 2 |
| ASM283484v1 | 6 | 4 | 4 | 5 | 2 | 5 | 3 | 2 |
| ASM283485v1 | 46 | 5 | 9 | 5 | 7 | 10 | 6 | 2 |
| ASM283487v1 | 5 | 5 | 3 | 5 | 2 | 5 | 3 | 2 |
| ASM283490v1 | 45 | 6 | 3 | 5 | 2 | 5 | 6 | 3 |
| ASM283492v1 | 43 | 6 | 4 | 15 | 2 | 5 | 3 | 3 |
| ASM283494v1 | 26 | 6 | 4 | 5 | 2 | 5 | 3 | 2 |
| ASM283495v1 | 42 | 15 | 13 | 13 | 2 | 12 | 6 | 2 |
| ASM283498v1 | 26 | 6 | 4 | 5 | 2 | 5 | 3 | 2 |
| ASM283504v1 | 40 | 6 | 11 | 11 | 2 | 12 | 3 | 2 |
| ASM283526v1 | 21 | 6 | 4 | 11 | 2 | 5 | 3 | 2 |
| ASM283528v1 | 52 | 16 | 3 | 5 | 7 | 17 | 6 | 2 |
| ASM283529v1 | 48 | 6 | 3 | 4 | 3 | 6 | 3 | 2 |
| ASM283530v1 | 6 | 4 | 4 | 5 | 2 | 5 | 3 | 2 |
| ASM283534v1 | 6 | 4 | 4 | 5 | 2 | 5 | 3 | 2 |
| ASM283536v1 | 49 | 6 | 8 | 5 | 7 | 5 | 14 | 2 |
| ASM283538v1 | 5 | 5 | 3 | 5 | 2 | 5 | 3 | 2 |
| ASM283540v1 | 47 | 6 | 3 | 5 | 7 | 5 | 6 | 2 |
| ASM283542v1 | 44 | 6 | 3 | 11 | 2 | 5 | 3 | 3 |
| ASM290266v1 | 4 | 3 | 3 | 4 | 3 | 4 | 3 | 3 |
| ASM325968v1 | 4 | 3 | 3 | 4 | 3 | 4 | 3 | 3 |
| ASM351748v1 | #4 | 3 | 3 | 4 | 3 | 4 | 17* | 3 |
| ASM435969v1 | #48 | 6 | 3 | 4 | 3* | 6 | 3 | 2 |
| ASM435971v1 | #7 | 6 | 3 | 6 | 3 | 7 | 3 | 3 |
| ASM435972v1 | #18 | 8 | 3 | 9* | 5 | 2 | 1 | 5 |
| ASM435973v1 | 28 | 11 | 3 | 5 | 7 | 8 | 3 | 3 |
| ASM1602879v1 | 4 | 3 | 3 | 4 | 3 | 4 | 3 | 3 |
| ASM1612707v1 | 8 | 6 | 3 | 5 | 3 | 7 | 3 | 3 |
| ASM1810774v1 | #33 | 6 | 17* | 10 | 3* | 2* | 17 | 12 |
| ASM1935751v1 | 47 | 6 | 3 | 5 | 7 | 5 | 6 | 2 |
| ASM1935755v1 | 60 | 6 | 6 | 7 | 6 | 10 | 5 | 6 |
| ASM767322v1 | 38 | 13 | 1 | 2 | 1 | 1 | 1 | 1 |
| ASM283500v1 | 41 | 15 | 3 | 11 | 2 | 5 | 3 | 3 |
| ASM283501v1 | 21 | 6 | 4 | 11 | 2 | 5 | 3 | 2 |

MLST marked with # was presented as the closest types.

Asterisks * indicate novel alleles with the closest types.

ND, not detect.
